# Report on the Prevalent Diseases of the City of Chicago, for the Last Two Months

**Published:** 1861-12

**Authors:** N. S. Davis

**Affiliations:** Member of the Sanitary Com.


### ARTICLE V.

### REPORT ON THE PREVALENT DISEASES OF THE CITY OF CHICAGO, FOR THE LAST TWO MONTHS.

By N. S. DAVIS, M. D., Member of the Sanitary Com.

Presented to the CHICAGO MEDICAL SOCIETY, Nov. 22d, 1861.

The last report was dated September 20, 1861. From that time to the 10th of October, I noticed no change in the health of the city, or in the character of the prevailing diseases. During the second week in October, the days were warm and sultry, while the nights were cool. During that time I noticed a decided increase in the number of attacks of diarrhœa, dysentery, and typhus fever, and yet neither of these diseases were more prevalent than is usual at that season of the year. During the second week in October, several cases of scarlet fever occurred in the North Division, in the neighborhood of Chicago avenue and Wolcott street. Six cases in that neighborhood came under my care during the second and third weeks of the month. Three of these proved to be mild throughout their course, but the other three presented severe anginose symptoms. The tonsils and lymphatic glands behind the angle of the jaw became rapidly much swollen and hard. The breathing became noisy from the rattling of tenacious mucus in the throat, the deglutition very difficult, the pulse frequent, and skin hot, dry, and covered thickly with the characteristic red rash. For these cases, I applied externally cloths over the swollen glands, constantly wet with an infusion of aconite leaves, in which was dissolved muriate ammonia. After this had been used thirty-six or forty-eight hours, I substituted the frequent application of a liniment of olive oil ℥ij., oil turpentine, ℥ss., and chloroform, ℥ss. Internally I directed the following, viz:

| | |
|---|---|
| ℞.—Chlorate Potassa, - - - - | ʒj. |
| Hydrochloric Acid, - - - | gttxx. |
| Tinct. Belladonna, - - - - | ʒj. |
| Water, - - - - - - - | ℥iij. |

Mix. Dose from 20 drops to a tea-spoonful, according to the age of the child, repeated every two hours. All these cases recovered without any unpleasant sequalæ. From that time to the tenth of the present month, I saw only three cases, all of a mild character. Since the tenth of this month, I have met with six or eight cases of this disease, in a neighborhood called "The Patch," lying between Old street and the Archer road, and between Arnold street and the south branch of the river. The inhabitants are almost exclusively Irish, and many of them living in shanties. In nearly all of these cases, the anginose symptoms were severe. The first case I saw, was a child about three years of age, Its skin was covered with the characteristic red rash, pulse 140 per minute; breathing short, hurried, and noisy from the rattling of tenacious mucus in the throat; the vessels of the conjunctiva were injected and eyes dull; the tonsils and palate were intensely red and much swollen; and the glands behind the angle of the jaw were also much swollen and hard. Deglutition appeared to be suspended, and the child was disposed to lie with the head thrown backward, without noticing anything around it. It died the following morning; doubtless, directly from mechanical obstruction to respiration. All the remaining cases I have seen in this neighborhood, have recovered, under the same treatment that was mentioned above. An incident occurred during the treatment of one of them, that may be worthy of mention. A little boy, six or seven years old, in the same family with the case that terminated fatally, was attacked suddenly and violently. I saw him about thirty-six hours after the commencement of the attack. The febrile action was severe; the rash had begun to show itself on the face and neck, and the fauces, tonsils, and glands behind the angle of the jaw were swelling rapidly. I directed cloths wet in the cool infusion of aconite leaves and muriate of ammonia, to be applied externally over the swollen glands; and an acidulated solution of chlorate of potassa, with tincture of belladonna, to be given at short intervals, internally. In about eighteen hours I was summoned to see him in great haste, with the representation that he was "out of his head, and dying." On arriving at the shanty, I found the boy with less fever, less frequent pulse, and slightly less swelling of the glands, and parts connected with the throat; but his upper eye-lids drooped as if partially paralyzed; his pupils were widely dilated, and eye-balls almost constantly rolling; frequent sudden startings and tossing of the hands; and a flighty or incoherent state of the mind. Believing all these *peculiar* symptoms to be the excessive effect of the belladonna, I directed the solution containing it, to be discontinued, and in its place, three grains of iodide of potassa, dissolved in half a drachm of aqua camphora, to be given every two hours, and a cloth wet in cold water, applied to the head. The next day all the symptoms of cerebral and nervous disturbance had disappeared, and what was still more gratifying, nearly all the *anginose* symptoms of the disease had also disappeared, and the patient made a more rapid recovery than any other one that had come under my care. From conversation with neighboring practitioners, I am led to believe that the scarlet fever has been quite prevalent for the last six weeks, in the extreme south part of the South and West divisions of the city. About the first of this month, cases of erysipelas began to occur in my practice, and in two weeks eight or ten cases had come under my care. Of these, three commenced in the face; one on the chest; one on the nates; two on the arms; and one at the verge of the anus. The two commencing on the arm and on the side, were of the phlegmonous variety, and were accompanied by suppuration and considerable gangrene of the sub-cutaneous cellular tissue. That upon the chest occurred in the person of a child only eighteen months old, which had been vaccinated on the arm, and the vaccine pustule was about three days advanced, when the erysipelas commenced on the same side of the chest, spread extensively, and was accompanied by extreme prostration and gangrene of the sub-cutaneous tissue. There was no appearance of erysipelas around the vaccine pustule, which passed through all its stages naturally. The child ultimately recovered. The other cases presented no unusual features, unless we include among them the following:

M. F., a native of Ireland, aged about thirty years, was suddenly attacked on the 21st of October, with a very acute and severe pain in the rectum. The patient was straining moderately at stool, when a sudden sharp, stinging pain was felt in the lower part of the rectum. It did not entirely cease, and in half an hour he went to stool again, when the pain became much aggravated and more continuous. In a few hours it became so intense, that he retired to bed and sent for me. I found him in bed writhing with pain, confined exclusively to the rectum, but without any fever or disturbance of the pulse, and without any swelling, redness, tenderness, or other sign of disease at the anus. To relieve the acute suffering, I directed the patient to take one-third of a grain of sulphate of morphia every two hours, and to keep a cloth closely applied to the anus, wet in a cold infusion of belladonna leaves. The next day I found the patient nearly relieved from pain, but complaining of much weakness. I again examined the anus carefully, and found no swelling, or other symptom of disease. The local narcotic application was continued, rest enjoined, and the internal use of the morphine omitted. The next day was passed comfortably, but on the 24th I was again called, and found the patient had had two evacuations from the bowels, and the pain, though by no means as acute as at first, was considerable. It had assumed a more dull character, became continuous, and was accompanied by moderate tenderness to firm pressure on the right of the anus. Still there was no redness or swelling externally, and only a very slight general febrile movement. Regarding it as probable that a cellular abscess was forming by the side of the rectum, I directed an emollient poultice externally, and anodynes internally, to allay pain and prevent too frequent evacuations from the bowels. The next day a slight degree of swelling and hardness was perceptible at the point of tenderness. The swelling continued slowly to increase on the right side of the anus, until the 28th, when it began to extend rapidly across the perineum, and on the morning of the 30th, it had involved the whole right half of the scrotum. Up to this time there was no erysipelatous redness upon the surface, and the swelling, everywhere, had a peculiar feel. It was soft, but neither fluctuating or crepitating. In twenty-four hours more, the swelling had extended over the whole scrotum and penis; the skin had become red and tense with several vesications, one of which was filled with bloody serum. Apprehending the near approach of extensive gangrene, although no distinct fluctuation existed, with the council of Dr. E. ANDREWS, one or two free incisions were made into the most prominent part of the scrotum, which gave exit to a large quantity of fœtid gas, and a very small amount of pus, which seemed to be diffused in the tissue, but nowhere collected into an abscess. During all the time, there was tendency to diarrhœa; a frequent, soft pulse; a coated and dry tongue; and occasional slight delirium. From the time the swelling began to extend across the perineum, to the 28th, the patient had been taking twenty drops of the tinct. ferri murias every four hours, with two grains of sulph. quinine and two of pulv. opii between. Notwithstanding these remedies and others of a supporting nature, gangrene extended rapidly throughout the skin and cellular tissue of the scrotum, penis, and perineum; thin and brown fœcal evacuations continued to occur; the tongue became brown and dry; the mind wandering, with subsultus, and weak but rapid pulse. Efforts were made to improve the condition of the intestinal mucous membrane, by an emulsion of oil of turpentine and tincture of opium, alternated with tannate of quinine, liquor ferri nitratis, etc., but without success. The gangrenous parts separated so rapidly, that by the 4th of November, the whole scrotum and a large part of the skin and cellular tissue of the penis had come off, leaving the testicles surrounded by the tunica vaginalis, and the spongy bodies of the penis fully exposed. During the fifth, sixth and seventh, the patient improved a little, and some granulations made their appearance on the surfaces from which the sloughs had separated. But on the morning of the 8th, a sudden and copious hemorrhage took place from the rectum, and the patient died in a few hours after. We have narrated this case, for the purpose of asking the Society the following questions, viz:

Was this a case of erysipelas commencing in the rectum, and extending, successively, to the skin and cellular tissue of the perineum, scrotum, and penis? or did the unhealthy and destructive inflammation result from a primary perforation of some point in the rectum, through which irritating and poisonous gases escaped into the cellular tissue, producing emphysema of the perineum, scrotum, etc.?

The patient had been troubled for several years, with, what he supposed to be, internal hemorrhoids. And from his last sickness, commencing with a sharp sting of pain in the act of defecation, from the fact that the mischief extended through the sub-cutaneous cellular tissue before involving the skin, and from the free escape of air from all the incisions, we are inclined to think the latter question suggests the true origin of the disease.

